# Supplementary material for: Adaptation and validation of the quality of contraceptive counseling (QCC) scale for use in Ethiopia and India
Source: PLoS One. 2023 Mar 31;18(3):e0283925. doi: 10.1371/journal.pone.0283925 (PMC10065231; doi:10.1371/journal.pone.0283925)
Supplement: Attachment — Submitted filename: Response to reviewer feedback 15Sep22.docx [file pone.0283925.s002.docx]

**Comments to the Author**

Reviewer #1: The manuscript is an interesting piece of work. It is well written; however, I would like to forward the the following points to further refine the manuscript.

1.) If you have an interest of comparing an underlying factor structures in the two countries, the manuscript didn't serve its purpose as multigroup CFA is missing from the manuscript. If otherwise, what is the point of separately reporting findings of the two countries without making an objective assessment (statistical comparison)?

- *Thank you for this clarifying question which helps us to refine the language we use in the manuscript to describe the QCC Scale versions. Our intention was not to compare the underlying factor structures between countries, but rather to assess independently the degree to which the two new scales* *replicated the factor structure and scale/item properties of the original QCC-Mexico scale. To make this more clear, we have made edits to the language throughout the entire manuscript to make clear that the QCC-Ethiopia and QCC-India scales were independently evaluated and that we consider these two separate, independently-assessed scales.*

2.) In line 125 to 126, it is indicated that clinic staffs are engaged in the process of sample selection and are aware of the study objective and its process in the study from Ethiopia. This more likely would have an impact on the result of the study (introducing bias) as providers may get to be at their best behavior during the study period.

- *Thank you for this point. We have added in line 414 an additional sentence to the Limitations: “*We also note that because providers in the study sites were aware the study was taking place, it is possible that they adjusted their behavior to provide better counseling; this could have inflated QCC scores*.”*

3.) In line 244-45, the authors mentioned that scores were skewed to higher quality and yet they used arithmetic mean as a summary measure which is highly influenced by skewed observation. Besides, the measurement is an ordinal scale and the use of arithmetic mean is not advisable in this scale of measurement.

- *Thank you for this helpful point. We have added information on the skew of scale and subscale scores to the Results and Table 3. Because D&A subscale scores were highly skewed, we re-ran the correlation analyses to use a dichotomous (top-scored) measure, as we had previously done in the validation study in Mexico (and updated Table 4 accordingly). We clarified in the Discussion that because of the skew of the D&A scores we recommend they be dichotomized when used as a standalone measure.*
- *Re: to the original line pointed out by this reviewer, in line 256 we have changed the word “skewed” to “tended” to more precisely represent the point, since we did not find evidence of skew other than that related to the D&A subscale.*
- *We have otherwise retained use of mean scoring per our understanding of the literature supporting use of mean scores for Likert scale data; see for example:*
  - *Sullivan GM, Artino AR Jr. Analyzing and interpreting data from likert-type scales. J Grad Med Educ. 2013 Dec;5(4):541-2. doi: 10.4300/JGME-5-4-18. PMID: 24454995; PMCID: PMC3886444.*
  - *Norman G. Likert scales, levels of measurement and the "laws" of statistics. Adv Health Sci Educ Theory Pract. 2010 Dec;15(5):625-32. doi: 10.1007/s10459-010-9222-y. Epub 2010 Feb 10. PMID: 20146096.*

4.) In line 252, the authors mentioned that they used MLVM command to run the factor analysis in STATA. MLVM is not a command; rather an option within the SEM command of STATA.

- *Thank you for this point; we have changed the word “command” to “option” accordingly.*

5.) In the reliability and validity analysis, the item "prov_insist" was kept as part of the third domain in Ethiopia and "opportunity" and "info_private" as part of the second domain. The reliability of these factors would have been improved if this items were removed. In addition, the items load poorly to their respective domains. This suggests that the authors should respecify the model and test a an alternative four or five factor CFA model (eg. pressure as a fourth domain) than retain then as part of the second or third.

- *Thank you for this suggestion. Per this reviewer’s first point, we have modified language to make more clear that our goal with this analysis was to examine the extent to which the QCC-Ethiopia and QCC-India Scales replicated the factor structure and scale/item properties of the original QCC-Mexico scale (rather than using exploratory factor analysis to determine the underlying factor structure of each). Because the QCC-Ethiopia and QCC-India scales contained only minor item modifications compared to the original QCC-Mexico Scale, we feel this approach is justified and that the utility of these adapted versions of the QCC Scale is higher with a consistent factor structure between countries such that the three QCC subscales can be applied in different settings.*

6.) The result section has a component that should have been taken to the discussion section (Lines 278 to 303).

- *Thank you for this helpful suggestion. We have moved this text to the discussion section –integrated into paragraphs 2-4—accordingly (with the exception of the sentence reporting findings from the model fit exercise which we deemed appropriate to retain in Results).*

Reviewer #2: The authors present a pertinent and well-founded justification for carrying out the investigation.
With regard to the structure of the article, it has a careful presentation, is written in English, easy to read, with a logical sequence and interconnection between the contents. Contains all mandatory elements for publications in PLOS ONE.
The methodology used fits the type of study developed. It presents all the steps that it integrates in a very detailed, clear and robust way. The inclusion and exclusion criteria of the participants, the type of and its calculation are explained. The tests used for the adaptation and validation of the Quality of Contraceptive Counseling (QCC) Scale for use in Ethiopia and India are adjusted and appropriate. The authors justify the robust way and, based on the statistical analysis performed, the inclusion of items on the scale (even those with a low factor), as well as the exclusion of others.
They present results of an original research and with high methodological rigor.
The discussion is well founded, supported by the data obtained through the results.
Conclusions lead us to the importance and relevance of using a valid and reliable scale as an instrument to be used in quality contraceptive counseling.
Most references are from the last 5 years.
The study respected the ethical principles required for studies with human beings: the authorizations of the University of California and the respective countries (Ethiopia and Egypt), as well as the informed consent given by the study participants.
I consider the article relevant for publication, due to the importance of dissemination, use of the scale and the methodological robustness presented.

- *Thank you for this assessment of our paper; we appreciate your review and feedback.*

**Journal Requirements:**

When submitting your revision, we need you to address these additional requirements.

1. Please ensure that your manuscript meets PLOS ONE's style requirements, including those for file naming. The PLOS ONE style templates can be found at 
[https://journals.plos.org/plosone/s/file?id=wjVg/PLOSOne_formatting_sample_main_body.pdf](https://urldefense.com/v3/__https:/journals.plos.org/plosone/s/file?id=wjVg*PLOSOne_formatting_sample_main_body.pdf__;Lw!!LQC6Cpwp!ov4L9A3AdyRDKXbgkvgXFMnNvC66jVyV-qPio4jOyXEQ2Hxgw6Yk6-y9L_NPpkH6-6lzSMzbdPlD6zofLCF3$) and 
[https://journals.plos.org/plosone/s/file?id=ba62/PLOSOne_formatting_sample_title_authors_affiliations.pdf](https://urldefense.com/v3/__https:/journals.plos.org/plosone/s/file?id=ba62*PLOSOne_formatting_sample_title_authors_affiliations.pdf__;Lw!!LQC6Cpwp!ov4L9A3AdyRDKXbgkvgXFMnNvC66jVyV-qPio4jOyXEQ2Hxgw6Yk6-y9L_NPpkH6-6lzSMzbdPlD63YsWNru$)

- *We have reviewed and confirmed formatting is correct.*

2. Please include a complete copy of PLOS’ questionnaire on inclusivity in global research in your revised manuscript. Our policy for research in this area aims to improve transparency in the reporting of research performed outside of researchers’ own country or community. The policy applies to researchers who have travelled to a different country to conduct research, research with Indigenous populations or their lands, and research on cultural artefacts. The questionnaire can also be requested at the journal’s discretion for any other submissions, even if these conditions are not met.  Please find more information on the policy and a link to download a blank copy of the questionnaire here: [https://journals.plos.org/plosone/s/best-practices-in-research-reporting](https://urldefense.com/v3/__https:/journals.plos.org/plosone/s/best-practices-in-research-reporting__;!!LQC6Cpwp!ov4L9A3AdyRDKXbgkvgXFMnNvC66jVyV-qPio4jOyXEQ2Hxgw6Yk6-y9L_NPpkH6-6lzSMzbdPlD67cUl6ON$). Please upload a completed version of your questionnaire as Supporting Information when you resubmit your manuscript.

- *Thank you for this innovative document. We have completed the file as requested and added additional detail to the Methods section on the approvals given by health center staff in each study site (page 4).*

3. In your Data Availability statement, you have not specified where the minimal data set underlying the results described in your manuscript can be found. PLOS defines a study's minimal data set as the underlying data used to reach the conclusions drawn in the manuscript and any additional data required to replicate the reported study findings in their entirety. All PLOS journals require that the minimal data set be made fully available. For more information about our data policy, please see [http://journals.plos.org/plosone/s/data-availability](https://urldefense.com/v3/__http:/journals.plos.org/plosone/s/data-availability__;!!LQC6Cpwp!ov4L9A3AdyRDKXbgkvgXFMnNvC66jVyV-qPio4jOyXEQ2Hxgw6Yk6-y9L_NPpkH6-6lzSMzbdPlD61283WAv$).

Upon re-submitting your revised manuscript, please upload your study’s minimal underlying data set as either Supporting Information files or to a stable, public repository and include the relevant URLs, DOIs, or accession numbers within your revised cover letter. For a list of acceptable repositories, please see [http://journals.plos.org/plosone/s/data-availability#loc-recommended-repositories](https://urldefense.com/v3/__http:/journals.plos.org/plosone/s/data-availability*loc-recommended-repositories__;Iw!!LQC6Cpwp!ov4L9A3AdyRDKXbgkvgXFMnNvC66jVyV-qPio4jOyXEQ2Hxgw6Yk6-y9L_NPpkH6-6lzSMzbdPlD69H7pgv7$). Any potentially identifying patient information must be fully anonymized.

Important: If there are ethical or legal restrictions to sharing your data publicly, please explain these restrictions in detail. Please see our guidelines for more information on what we consider unacceptable restrictions to publicly sharing data: [http://journals.plos.org/plosone/s/data-availability#loc-unacceptable-data-access-restrictions](https://urldefense.com/v3/__http:/journals.plos.org/plosone/s/data-availability*loc-unacceptable-data-access-restrictions__;Iw!!LQC6Cpwp!ov4L9A3AdyRDKXbgkvgXFMnNvC66jVyV-qPio4jOyXEQ2Hxgw6Yk6-y9L_NPpkH6-6lzSMzbdPlD60uLkGXw$). Note that it is not acceptable for the authors to be the sole named individuals responsible for ensuring data access.

We will update your Data Availability statement to reflect the information you provide in your cover letter.

- *We have added the data to Dryad and added the link in our revised cover letter*

4. We note that you have stated that you will provide repository information for your data at acceptance. Should your manuscript be accepted for publication, we will hold it until you provide the relevant accession numbers or DOIs necessary to access your data. If you wish to make changes to your Data Availability statement, please describe these changes in your cover letter and we will update your Data Availability statement to reflect the information you provide.

- *We have added the data to Dryad and added the link in our revised cover letter*

5. Please note that in order to use the direct billing option the corresponding author must be affiliated with the chosen institute. Please either amend your manuscript to change the affiliation or corresponding author, or email us at [plosone@plos.org](mailto:plosone@plos.org) with a request to remove this option.

- *Kelsey Holt, corresponding author, information is up-to-date with University of California as the affiliated institution*

6. PLOS requires an ORCID iD for the corresponding author in Editorial Manager on papers submitted after December 6th, 2016. Please ensure that you have an ORCID iD and that it is validated in Editorial Manager. To do this, go to ‘Update my Information’ (in the upper left-hand corner of the main menu), and click on the Fetch/Validate link next to the ORCID field. This will take you to the ORCID site and allow you to create a new iD or authenticate a pre-existing iD in Editorial Manager. Please see the following video for instructions on linking an ORCID iD to your Editorial Manager account: [https://www.youtube.com/watch?v=_xcclfuvtxQ](https://urldefense.com/v3/__https:/www.youtube.com/watch?v=_xcclfuvtxQ__;!!LQC6Cpwp!ov4L9A3AdyRDKXbgkvgXFMnNvC66jVyV-qPio4jOyXEQ2Hxgw6Yk6-y9L_NPpkH6-6lzSMzbdPlD6-oeKYP0$)

- *I have linked to my ORCID (Kelsey Holt)*

7. Please include your full ethics statement in the ‘Methods’ section of your manuscript file. In your statement, please include the full name of the IRB or ethics committee who approved or waived your study, as well as whether or not you obtained informed written or verbal consent. If consent was waived for your study, please include this information in your statement as well.

- *Thank you for pointing out this omission. We have added additional detail on Pg 5 in the Ethics statement section on the use of oral consent.*

8. Please include captions for your Supporting Information files at the end of your manuscript, and update any in-text citations to match accordingly. Please see our Supporting Information guidelines for more information: [http://journals.plos.org/plosone/s/supporting-information](https://urldefense.com/v3/__http:/journals.plos.org/plosone/s/supporting-information__;!!LQC6Cpwp!ov4L9A3AdyRDKXbgkvgXFMnNvC66jVyV-qPio4jOyXEQ2Hxgw6Yk6-y9L_NPpkH6-6lzSMzbdPlD61Wg8s5y$).

- *Thank you. We added a caption list after acknowledgements, on pg. 24*
