# Supplementary material for: Adaptation and validation of the quality of contraceptive counseling (QCC) scale for use in Ethiopia and India
Source: PLoS One. 2023 Mar 31;18(3):e0283925. doi: 10.1371/journal.pone.0283925 (PMC10065231; doi:10.1371/journal.pone.0283925)
Supplement: Attachment — Submitted filename: Response to reviewer comments March 3 2023.docx [file pone.0283925.s003.docx]

Reviewer #1: The authors confused the use of exploratory factor analysis with confirmatory factor analysis. In one hand, they report that the aim of their work is tool validation which was supposed to be done via confirmatory analysis. Yet, the strategy utilized during their analysis was a typical characteristics of an exploratory analysis.

**Thank you for this comment. We have in fact conducted a confirmatory analysis, as detailed in the Analysis section beginning line 169.**

Reviewer #2: (No Response)

Reviewer #3: This is a thorough, well-done study. The reporting is clear. The inclusion of two different countries in the survey offers added value.

I have the following questions/comments:
1. Is there any information available about the response rate?

**No, we do not have response rates available. Sampling was done by convenience sampling, as detailed in the “Sample, recruitment, and data collection” section starting on line 117.**

2. The factor analysis reveals that the QCC consists of 3 subscales. Does it make sense to calculate an overall QCC score (and an overall Cronbach's alpha)?

**Thank you for this question. We argue that the Pearson correlation coefficients between subscale scores are high enough to suggest a single unifying latent QCC construct—as was similarly observed in the published Mexico version of the scale. The paragraph beginning line 308 provides the following detail:**

**“Examination of the Pearson correlations coefficients between the subscale scores showed that the relationships observed in Mexico largely remained the same in Ethiopia, providing evidence for a single unifying latent QCC construct in this setting; specifically, the Information Exchange subscale and Interpersonal Relationship subscales were highly correlated (0.8 in Ethiopia, similar to the 0.7 observed in Mexico) and the Disrespect and Abuse subscale correlated 0.3 with both Information Exchange and Interpersonal Relationship in Ethiopia compared to the 0.3 and 0.4, respectively, observed in Mexico [11]. In India, the Information Exchange and Interpersonal Relationship subscales were correlated similarly to the other two countries (0.7); however, the Disrespect and Abuse subscale was less correlated with the other subscales (0.1 with Information Exchange and 0.05 with Interpersonal Relationship).”**

**In the Discussion, we provide further guidance, noting the caveat that in India the D&A subscale was less correlated with the other subscales:**

**“It is also worth noting that the Disrespect and Abuse subscale was less correlated with other subscales in our India sample compared to the Ethiopia sample and what had previously been observed in the QCC-Mexico validation study. This suggests that in the Vadodara context, the experience of Disrespect and Abuse is more weakly tied to the overall QCC construct; in other words, a person’s experience of extreme negative provider behaviors is less correlated with how they perceived counseling overall compared to other settings. Nonetheless, noting this limitation, composite scores can still be computed and applied to capture the full range of the QCC construct.”**

**Further, the individual subscale alphas are also provided in Table 3.**

3. The item prov_insist is clearly measuring slightly differently from the other items in the "disrespect and abuse" subscale. I suggest to remove this item from the subscale. Since this item is considered important for the content validity, this item can still be included in the questionnaire, but it should not be used to calculate the score for “the disrespect and abuse” subscale.

**Thank you very much for this observation. It has motivated us to modify the analysis and move this item to the IR subscale where it performs much better. Because disrespect and abuse is highly related to the interpersonal relationship between provider and client, this change is justified conceptually. Line 276- footnote to Table 3-now reads:**

**“This item [prov_insist] loaded as part of the Disrespect and Abuse subscale in the original scale development in Mexico [11]. In our CFA modeling it performed very poorly on that subscale (subscale alphas were 0.4 and 0.6 and the item loaded 0.02 and 0.14 in Ethiopia and India, respectively) and thus was moved to the conceptually-related Interpersonal Relationship subscale, as shown here”**

**Including the item in the IR subscale greatly improved the alphas for the D&A subscale in both countries and the loading of the item in India (though not Ethiopia, where it was nonetheless retained due to the critical importance of capturing potential pressure from providers from a content validity perspective). This change did not qualitatively change interpretation of findings related to goodness of fit or correlational validity, though the values were updated in the Results (Tables 3 & 4, respectively).**

**Other changes made:**

- **We caught a typo on line 100 about the length of the cognitive interviews in Ethiopia and updated to indicate that they were, on average, 30 minutes**
- **The QCC Website containing the item wording has been updated; accordingly, we replaced the old url with the new one in the text of the manuscript:** <https://qccscale.ucsf.edu/qcc-scale-versions>
- **In the Discussion, we referenced the short version of the QCC scale (the QCC-10); two new articles have been published so we added those citations to the reference section (19-20)**
